# Radiofrequency VS Cold Surgery to Treat Oral Papillomatous Lesions

**DOI:** 10.22038/ijorl.2020.38177.2256

**Published:** 2021-03

**Authors:** Bruno Galletti, Francesco Gazia, Cosimo Galletti, Francesco Freni, Cosimo Galletti, Rocco Bruno, Federico Sireci, Francesco Galletti

**Affiliations:** 1 *Department of Otorhinolaryngology,* * A.O.U.P. Paolo Giaccone, Via del Vespro, 129, 90127 Messina, Italy* *.*; 2 *Department of D* *entistry, University of Barcelona, Barcelona, Spain.*; 3 *Department of Otorhinolaryngology,* * A.O.U.P. Paolo Giaccone, Via del Vespro, 129, 90127 Palermo, Italy* *.*

**Keywords:** Benign Neoplasms, Human Papilloma Virus, Oral Cavity, Radio Frequency Ablation

## Abstract

**Introduction:**

The aim of this study is to analyse different surgery (radio frequency and cold instrumentation) of oral benign papillomatous lesions.

**Materials and Methods:**

A retrospective study was carried out in our section of Otorhinolaryngology from 2014 to 2018. 112 patients with oral benign papillomatous lesions were enrolled and divided into 2 groups. Group A of 62 patients treated with excision of lesions using radio frequency using a bipolar coagulation electrode (CelonLabENT). Group B of 50 patients treated with excision of the lesion using traditional cold instruments (scalpel and surgical forceps). All patients were evaluated for intraoperative bleeding, discomfort and recurrence rate.

**Results:**

112 patients (of which 37 males and 75 females) with mean age 32.9 ranged from 10 to 61 years. The HPV types associated with oral benign papillomatous lesions were HPV 6 (17%), 11 (23,3%), 13 (10,7%), 32 (34%), 2 (10%) and 57 (5%). There are no statistically significant differents among patients operated with radio frequency (Group A) and cold instrumentation (Group B) regarding intraoperative bleeding (P= 0.08), recurrence rate after 1 year from surgery (P=1), intraoperative discomfort (P=0.7) and postoperative discomfort (P=0.6).

**Conclusion:**

Radiofrequencies and surgery with scalpel and surgical forceps are equal valid methods for the treatment of benign papillomatous.

## Introduction

In the Papilomavaviridae family, Human papillomavirus (HPV) are the most frequent virus relating to human popolation. HPV are small viruses with a genome of 8.1 kilobases in double circular DNA. The frequency of HPV in oral mucosa without lesions ranges from 0.7% to 82%. Oral squamous papilloma, oral verruca vulgaris, oral condyloma accuminatum and focal epithelial hyperplasia are the typically HPV benign papillomatous lesions. 

They appear classically in the oral mucosa. These lesions are classically asymptomatic or occur with a foreign body sensation in the oral cavity (1-6). 

The differential diagnosis between oral verruca vulgaris, oral squamous papilloma, focal epithelial hyperplasia and oral codylomaaccuminatum in the oral mucosa may be done with inspection of the HPV lesion and also with biopsy and cytology. 

Squamous cell papilloma (SCP) is an uncommon single benign epithelial neoplasm of the oral cavity. SCP is a lesion without pain which can occur at any oral place. Usually SCP is characterized by an excessive keratinization and the lesion appears whitish. Condylomaaccuminatum (CA) is predominantly seen on the skin and mucosal surfaces of the anogenital tract. CA is characterized by a mulberry-like or cauliflower-like form. Heck’s disease is the classic name of the Focal epithelial hyperplasia (FEH) that typically interested the children (7-8). FEH lesions are different no-symptomatic wound of classic rose colour. 

Verruca vulgaris (VV) is a lesion of the skin that interested different part of the body, for example mucosal surfaces of the genito-anal region, fingers, feet, face, back of the hand and eyelids. Oral interest is uncommon. The lesions of oral cavity typically result from autoinoculation of the virus through the fingers. 

Thanks to molecular biology exams, it is possible to research the HPV DNA in the lesion. The polymerase chain reaction is the most sensitive and specific method to find HPV. Various types of HPV of oral benign lesions are described in the literature. 

A prevalence of HPV 6 and 11 was found in SCP and CA, while HPV 2 and 57 were more frequent in VV. The prevalence of HPV 13 and 32 was observed in FEH. In this study, surgery was performed using a radiofrequency system in Group A. Radiofrequency causes ionic movement in cells, creatng hypertermic necrosis of the mucosa. The classic temperature can be conditioned from 59°C to 76°C to avoid necrosis of the healthy tissue close up. Group B was treated using traditional cold instruments (scalpel and surgical forceps).

The aim of this study is to analyse surgery of benign papillomatous lesions of the oral cavity among patients operated with radio frequency and traditional cold instrumentation.

## Materials and Methods

A retrospective study was carried out in our ENT section from 2014 to 2018. We have included in our study only HPV-DNA positive patients. Biopsy was used for all lesions for definite diagnosis.

112 patients with oral benign papillomatous lesions were enrolled. All the patients underwent detailed clinical examinations about the size, the site, the number, the shape, the colour, the consistency of the lesions.

Polymerase Chain Reaction (PCR) was used to find the HPV DNA in lesions. 

The patients were divided in two groups based on surgical modality. Group A consists of 62 patients treated from 2015 to 2017 with excision of lesions usingradio frequency using a 15-W bipolar coagulation electrode (Celon Lab ENT).

Group B consists of 50 patients treated from 2013 to 2014 with excision of the lesion using traditional cold instruments (scalpel and surgical forceps), because in our clinic there was no radio frequency available.

All patients were treated under local anesthesia with mepivacaine 20 mg/ml with adrenaline 1:100.000 injected at the perilesional level. 

For all patients were evaluated intraoperative bleeding, recurrence rate after 1 year, Visual Analogue Scale (VAS) about intra and post-operative discomfort. Statistical analyses were done using SPSS 24.0 (IBM SPSS Statistics). The data are presented as means with standard deviations. 

Data normality was valuated using the Kolmogorov-Smirnov test of normality. The Man-Whitney-U-Test was used to analyze measurements of VAS. The Fisher exact test was used for evalueting the percentage of bleeding and the recurrence rate. A P ≤ 0.05 was been considered signicant. 

## Results

112 patients of which 37 males and 75females with mean age 32.9 ranged from 10 to 61 years were included the study. The HPV types associated with oral benign papillomatous lesions were HPV 6 (17%), 11 (23,3%), 13 (10,7%), 32 (34%), 2 (10%) and 57 (5%).

The types of lesions are shown in Table 1.

**Table 1:** The types of lesions in two groups

	**Cases**	**Age**	**Sites**	**Size**	**Number**	**HPV Types**
SCP	12(10.7%)	44.2 (24- 61)	Soft palate (50%), tongue (40%), upper lip (10%)	0.8-1.3	Solitary	6 (33%); 11(66%)
FEH	50 (44%)	18.3 (10-28)	Tongue (20%), lower lip (75%), alveolar mucosa (5%)	0.2-0.8	Multiple	13 (24%); 32(76%).
VV	17 (15.2%)	32.5 (19-54)	Lips (63%), hard palate (26%), tongue dorsum (11%)	0.7-1.8	Multiple	2 (65%); 57 (35%)
CA	33 (29.5%)	36.6 (22-58)	The tongue (32%), palate (56%), lower lip (22%)	0.5-2.7	Solitary/Multiple	6 (45%); 11(55%)

*SCP* Squamous Cell Papilloma, *FEH* Focal Epithelial Hyperplasia, *VV* Verruca Vulgaris, *CA* Condyloma Accuminatum

There are no statistically significant differences between sex, age, lesion type and HPV genotype between the two groups. There are no statistically significant differences among patients operated with radio frequency (Group A) and cold instrumentation (Group B) regarding intraoperative bleeding (P=0.08), recurrence rate after 1 year from surgery (P=1), VAS about intra (P=0.7) and postoperative discomfort (P=0.6) (Table.2). 

**Table 2:** Factors evaluated in two groups expressed as Mean ± Standard Deviation

	**Group A**	**Group B**	**P value**
Bleeding	0 (0%)	1 (2%)	0.8
Recurrence Rate	1 (1.7%)	1 (2%)	1
VAS intraoperative	2.66 ± 1.1	2.76 ± 1.32	0.7
VAS postoperative	2.97 ± 1.2	2.8 ± 0.9	0.6

## Discussion

Diagnosis of papillomatous lesion in the oral cavity and the oropharynx may be done thanks to clinical examination and biopsy (9). 

Dona et al. publicated in 2017 a review about the anatomical distribution of different HPV types diagnosed in oral benign papillomatous lesions. The prevalence of mucosal HPV infections in this article was 28.9 %, half compared to our study,instead, the distribution of anatomical sites is very similar to our cases(10).

Actually, in the scientific literature, the treatment of choice for oral squamous papillomas is the surgical excision, that can be done using cold-steel excision, electrocautery, laser, intralesional injections of interferon or cryosurgery. There is no a high recurrence rate for the multiple lesions compared with solitary type, only in patients with Human Immunodeficiency Virus (HIV) recurrence is higer (11). The laser assisted surgery has several advantages, as haemostasis, high precision in tissue destruction, wound sterilization, devoid of sutures, edema and minimal post-operative pain. In consideration of these advantages, several cases report use diode laser surgery reporting no pain medication after excision, wound healing notable and rapidly achievable, in addition may provide tolerable procedure for paediatric patients to remove oral lesions (12,13). Cryosurgery is a technique characterized by the necrosis of affected or healty cells and tissues, with the action of the cold elements at sub-zero degree. The advantages of this treatment are absence of bleeding, minimum need for anaesthesia and a faster and less traumatic postoperative period (14). In our study, surgery was performed using a radiofrequency system in Group A. Radiofrequency causes ionic movement in cells, creatng hypertermic necrosis of the mucosa. The classic temperature can be conditioned from 59°C to 76°C to avoid necrosis of the healthy tissue close up (15-17). Group B was treated with cold instrument surgery. In our study, it is noted that radiofrequencies and surgery with cold instruments are valid methods for the treatment of benign papillomatous lesions. No statistically significant results were observed regarding intraoperative bleeding, recurrence rate after 1 year from surgery, VAS about intra and postoperative discomfort. Our opinion is that all methods of surgical excision can be decisive for the treatment of benign papillomatosis (18,19). 

As for medical therapy, there are many manuscripts in the literature on the use of the antiviral cidofovir in papillomatous lesions. This antiviral seems to have excellent results on the remission of the pathology, avoiding relapses. Cidofovir has only been used in laryngeal pathology. Until now, there are no manuscripts on the use of local antiviral injections in the oral cavity, further studies would be needed (20-22).

The main purpose of surgery is to completely remove the lesion, also removing part of healthy tissue to prevent recurrences.

Looking at recent work in the literature, the prevalence of the various types of papillomatous lesions of the oral cavity, obtained in our results, is comparable to the results found in the literature. Regarding the various types of treatment, ours is the first work in the literature that compares the results of two different surgical techniques. All the manuscripts that describe the use of radiofrequencies or the traditional scalpel are free of local complications such as bleeding and no recurrence, so the results are similar to those of our study (1,9,14,23).

More studies in the literature would be useful to compare the various surgical methods, because to date the methods of treatment are described only in some case reports.

## Conclusion

Based on result of this study, radiofrequencies and surgery with cold instruments are equally effective methods for the treatment of benign papillomatous. The choice of surgical method is mainly based on the experience of surgeons and the availability of instruments when dealing with these lesions
